# DEMENTIA CARE SHARED BY MULTIPLE CHINESE FAMILY CAREGIVERS: EXPLORING FAMILY-LEVEL PREPAREDNESS FOR CAREGIVING

**DOI:** 10.1093/geroni/igae098.4155

**Published:** 2024-12-31

**Authors:** Jacky C P Choy, Dipsy H S Wong

**Affiliations:** The University of Hong Kong, Hong Kong, China (People’s Republic); The University of Hong Kong, Hong Kong, China (People’s Republic)

## Abstract

Involvement of multiple family members as caregivers is commonly observed and often essential in families living with dementia. While preparedness for caregiving was found to be associated with better caregiver outcomes, previous studies centred on one primary caregiver and rarely enquired their preparedness at a family level. This qualitative study aims to explore the preparedness of family as a unit for dementia care. Participants were 30 caregivers (12 spousal caregivers; 31 to 86 years old) who took care of a person living with dementia (PLwD) in the community and reported having more than one family members involved (3.8 caregivers involved in average). We conducted semi-structured in-depth interviews to understand the care needs of PLwD, their care arrangements, family dynamics, and perceived family preparedness. Thematic analysis with an inductive approach was adopted. Seven themes suggesting 7 components of family preparedness emerged: 1) Common understanding of PLwD care needs, 2) Aligned caregiving roles and expectations, 3) Identification of primary caregiver, 4) Flexibility for care task sharing and substitution, 5) Conflict resolution in care arrangement decision making, 6) Exchange of caregiving resources and information, and 7) Sharing of feelings and emotional support. Proximity, composition, family values, and prior communication patterns among members substantially shape their preparedness for caregiving. These components largely resonate with the dimensions of communication, roles, problem solving and affective exchange in McMaster model of family functioning and resemble concepts in models of team effectiveness. Family-centered interventions targeting family preparedness for caregiving can potentially improve caregiver as well as family outcomes.

